# Elucidating the role of disorder and free-carrier recombination kinetics in CH_3_NH_3_PbI_3_ perovskite films

**DOI:** 10.1038/ncomms8903

**Published:** 2015-07-30

**Authors:** Chan La-o-vorakiat, Teddy Salim, Jeannette Kadro, Mai-Thu Khuc, Reinhard Haselsberger, Liang Cheng, Huanxin Xia, Gagik G. Gurzadyan, Haibin Su, Yeng Ming Lam, Rudolph A. Marcus, Maria-Elisabeth Michel-Beyerle, Elbert E. M. Chia

**Affiliations:** 1 Division of Physics and Applied Physics, School of Physical and Mathematical Sciences, Nanyang Technological University, 21 Nanyang Link, Singapore 637371, Singapore; 2 School of Materials Science and Engineering, Nanyang Technological University, Singapore 639798, Singapore; 3 Noyes Laboratory, California Institute of Technology, Pasadena, California 91125, USA

## Abstract

Apart from broadband absorption of solar radiation, the performance of photovoltaic devices is governed by the density and mobility of photogenerated charge carriers. The latter parameters indicate how many free carriers move away from their origin, and how fast, before loss mechanisms such as carrier recombination occur. However, only lower bounds of these parameters are usually obtained. Here we independently determine both density and mobility of charge carriers in a perovskite film by the use of time-resolved terahertz spectroscopy. Our data reveal the modification of the free carrier response by strong backscattering expected from these heavily disordered perovskite films. The results for different phases and different temperatures show a change of kinetics from two-body recombination at room temperature to three-body recombination at low temperatures. Our results suggest that perovskite-based solar cells can perform well even at low temperatures as long as the three-body recombination has not become predominant.

## Abstract (web summary)

In solar cells, the density and the mobility of charge carriers govern the device performance. Here the authors determine these quantities independently in perovskite films by time-resolved terahertz spectroscopy to study the influence of disorder and crystal structure as a function of temperature.

## Graphical abstract

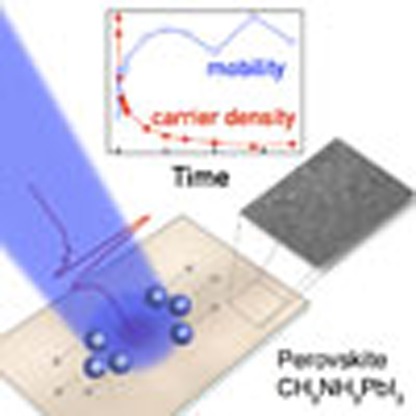

The high efficiency of the organometallic lead halide CH_3_NH_3_PbI_3_ and other related perovskites as photoactive components in photovoltaic devices caused huge excitement in the solar cell community. The material demonstrates promising solar-conversion efficiency exceeding 15%, resulting from the combined advantages of broadband absorption across the solar spectrum together with high carrier yields and mobilities^1^,^2^,^3^,^4^,^5^.

In methylammonium lead iodide CH_3_NH_3_PbI_3_ (referred to throughout this paper as perovskite), on excitation at energies that significantly exceed the lowest exciton transition, hot electrons and holes are created followed by thermalization via fast cascading^6^ into bound and/or free carriers at the conduction and valence band edges. The subsequent dynamics is determined by the interplay between the density and mobility of the free carriers in the sample, which manifests itself as complex optical conductivity spectra at different times after photoexcitation. It is therefore desirable to perform a time-resolved experiment to monitor the time evolution of the free carriers, and from it to independently determine their density and mobility.

Terahertz radiation (1 THz=4.1 meV) probes free carriers via their direct interaction with the terahertz electric field. Using terahertz pulses, we can perform time-resolved terahertz spectroscopy (TRTS)^7^, which probes the free-carrier dynamics via the photoinduced change in the conductivity (photoconductivity), as a function of time delay after photoexcitaton. TRTS allows for the direct determination of both real (Δ*σ*_1_) and imaginary (Δ*σ*_2_) photoconductivity without the need for the Kramers–Kronig transformation. Knowing both the real and imaginary parts simultaneously restricts the theoretical models that can explain the data.

Previous TRTS studies of thin perovskite films on quartz and on mesoscopic supports^8^,^9^,^10^ focus on the room-temperature dynamics of free carriers obtained by measuring frequency-integrated photoconductivity. From those measurements, only lower bounds of carrier mobilities were obtained. In contrast, in our present work, we make use of the full potential of TRTS by performing a spectral analysis of Δ*σ*_1_(*ω*) and Δ*σ*_2_(*ω*) to analyse the photoconductivity covering a broad terahertz range from 0.5 to 2.5 THz, as a function of temperature from 15 to 285 K, where a tetragonal-to-orthorhombic structural phase transition at 162.5 K has been reported for single crystals of this material^11^.

For the perovskite films studied in this paper, our analysis reveals a significant contribution from backscattering of charge carriers due to disorder. Analysis of the time evolution of the carrier density via a rate equation allows us to extract the absolute values of the rate constants and not only their lower bounds^8^. The observed recombination dynamics revealed a third-order kinetics at low temperatures, which is attributed to an Auger mechanism. On increasing the temperature, this mechanism crosses over to a second-order recombination mechanism near room temperature, regarded as a conventional electron transfer reaction. From the rate constants, we estimate the diffusion length of charge carriers at 285 and 180 K to be close to 1 μm, thus favouring this perovskite as a multitasking light converter in a solar cell.

## Results

### Time-resolved terahertz spectroscopy of perovskite

**Figure 1:**
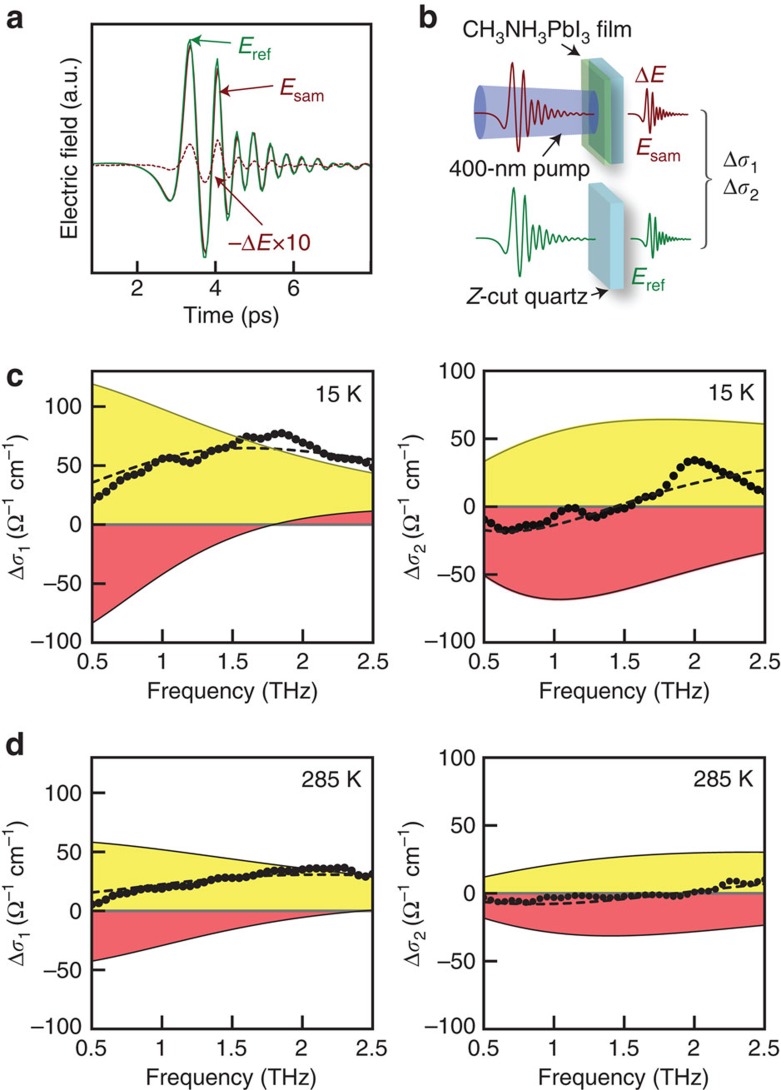
Terahertz transient and photoconductvity spectra. (**a**,**b**) Terahertz transient of the CH_3_NH_3_PbI_3_ thin film after optical excitation with 400-nm pump pulses with a fluence of 27 μJ cm^−2^. The photoconductivity is calculated from the terahertz transmitted electric field through the perovskite film (*E*_sam_), the z-cut quartz reference (*E*_ref_) and the electric field transient (Δ*E*). The complex terahertz photoconductivity (Δ*σ*_1_+*i*Δ*σ*_2_) obtained at 4.5 ps at (**c**) 15 K and (**d**) 285 K shows contributions from Drude (yellow), Smith (pink) and total (dashed) terms. The Smith term arises from the sample disorder and contributes both a downturn in Δ*σ*_1_ and a negative Δ*σ*_2_ at the lowest frequencies.

In our experiments, films of nanocrystalline CH_3_NH_3_PbI_3_ (thickness ∼230 nm) on z-cut quartz are grown by a standard solution-based single-step protocol (see Methods). The perovskite sample is excited by 400-nm (3.1 eV) optical pump pulses that contain about 1.5 eV excess energy above the exciton threshold located at 750 nm. After a well-defined pump-probe time delay *τ*, a terahertz probe pulse arrives at the sample. From the transmitted terahertz electric field *E*(*t*,*τ*) and the photoinduced change in terahertz electric field Δ*E*(*t*,*τ*), we extract the complex photoinduced change in the complex optical conductivity 
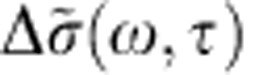
 (referred to as photoconductivity throughout this paper) as a function of *τ* (see Fig. 1a,b). More detailed descriptions of sample preparation, sample morphology, device performance, temperature-dependent absorption spectra, experimental set-up, TRTS data analysis and additional data can be found in the Supplementary Discussion and Supplementary Methods.

The real [Δ*σ*_1_(*ω*)] and imaginary [Δ*σ*_2_(*ω*)] parts of the complex photoconductivity, at *τ*=4.5 ps after photoexcitation are compared in Fig. 1c (at 15 K) and Fig. 1d (at 285 K). The time evolutions of Δ*σ*_1_(*ω*) and Δ*σ*_2_(*ω*) at 15, 80, 180 and 285 K depicted in Supplementary Fig. 2 show that Δ*σ*_1_(*ω*) decreases with increasing time delay after photoexcitation, implying that the free carriers are decaying.

### Drude–Smith analysis to account for sample disorder

In the Drude model of free carriers, at the lowest frequencies, Δ*σ*_1_(*ω*) increases with decreasing frequency and reaches a maximum at zero frequency, while Δ*σ*_2_(*ω*) should be zero. However, in our data Δ*σ*_1_(*ω*) increases with increasing frequency, while Δ*σ*_2_(*ω*) is negative at low frequencies. This observation is consistent with the influence of disorder on the free-carrier dynamics^12^. In the Drude–Smith model, the complex photoconductivity 
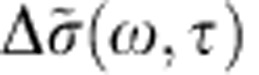
 is the sum of Drude and Smith conductivities^13^:


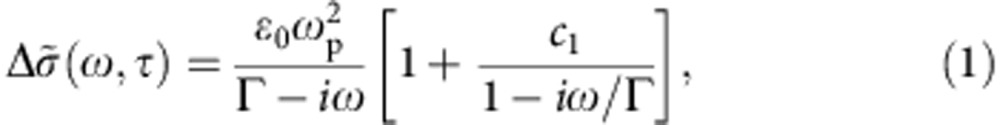


where the first term 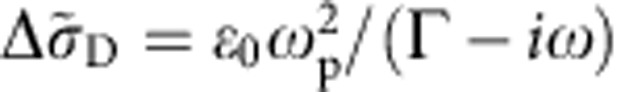 is the Drude conductivity, while the second Smith term 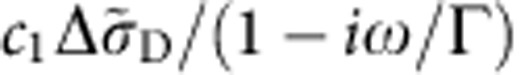 modifies the Drude model by accounting for the backscattering of carriers (*c*_1_ is negative) presumably off grain boundaries or defects that are expected in these perovskite films. The Drude–Smith model consists of three free parameters: *ω*_p_, Γ and *c*_1_, where *ω*_p_ is the Drude plasma frequency related to the free-carrier density via 
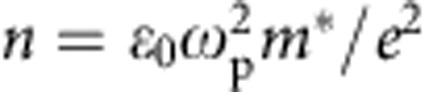
, with m*=0.2*m* being the effective electron mass^14^, *ɛ*_0_ is the vacuum permittivity and *e* is the electron charge. Γ denotes the scattering rate of the free carriers, which is related to carrier mobility via the expression *μ*=e/(Γm*). The parameter *c*_1_, defined as the fraction of initial velocity of carriers after a collision, describes the strength of backscattering due to localization, and ranges between −1 (complete backscattering) and 0 (no backscattering)^13^. The Drude–Smith fits to the data are displayed as dashed lines in Fig. 1c,d and in Supplementary Fig. 2. The broad peaks in Supplementary Fig. 2 near 2 THz reflect the pump-induced terahertz response due to the coupling between the charge carriers and the phonons in CH_3_NH_3_PbI_3_, that were not compensated by the reference (substrate) signal^10^.

### Extracting carrier mobility and its dynamics

**Figure 2:**
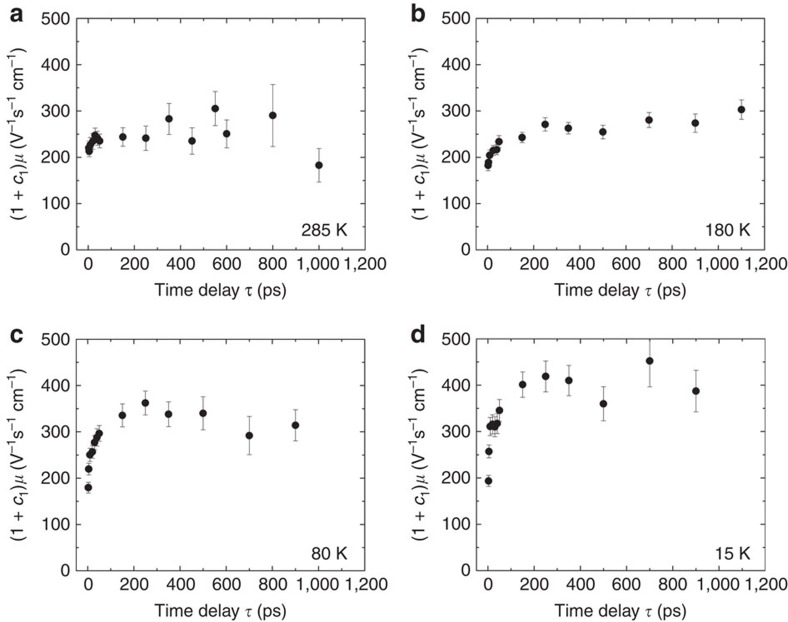
High free-carrier mobility of perovskite. Disorder-influenced free-carrier mobility obtained from the Drude–Smith fits is plotted against pump-probe time delay *τ*, for (**a**) 285 K, (**b**) 180 K, (**c**) 80 K and (**d**) 15 K. Mobility rises initially, then flattens off at long time delays. The error bars of the data points are the standard error of the mean obtained by fitting the complex conductivity spectra to the Drude–Smith model.

The Drude–Smith fits (shown in Fig. 1c,d and Supplementary Fig. 3) yield the time evolution of the free-carrier mobility. The existence of the Smith term reduces the d.c. conductivity 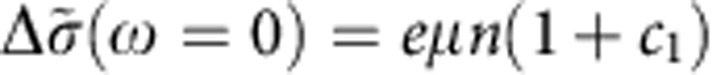 by a factor (1+*c*_1_), and hence the carrier mobility according to the Drude–Smith model becomes (1+*c*_1_)*μ*. Figure 2 shows the time dependence of (1+*c*_1_)*μ*(*τ*). At all temperatures, we see an initial increase in the mobility up to ∼50 ps, which then gradually flattens off. The initial increase of the mobility can be attributed to the initial cooling of hot carriers^12^, which was also observed in the doped InSb semiconductor^15^. This TRTS method can be used for comparative studies of the free-carrier mobility in perovskite films with different morphology when grown under different conditions, such as from solutions following the single-step^3^,^16^ or two-step method^4^, by vapour deposition^17^, or on various supports (for example, films on semiconductor oxides as mesoporous alumina or on a flat surface as quartz). The potential interplay between defect absorption and carrier mobility is of particular interest for single perovskite crystals that have been shown in experiment and calculation to have a pronounced discontinuity in the absorption spectrum between 750 and 500 nm^18^.

### Rate equation analysis of recombination kinetics

The same Drude–Smith fits also yield the time evolution of the free-carrier density *n*(*τ*). For the description of *n*(*τ*), we use the rate equation (Equation 2) to analyse the dynamics that is attributed to the recombination of charge carriers:


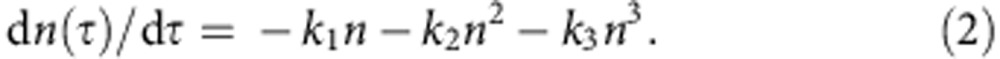


Equation (2) involves multiple-order recombination mechanisms providing different channels for the decrease of free-carrier density. Our fit contains four fitting parameters: the initial carrier density *n*_0_ and the three kinetic parameters *k*_*i*_. The rate constant *k*_1_ corresponds to the removal of either carrier to form an immobile species (for example, by trapping), *k*_2_ describes a bimolecular recombination that requires two distinct bodies and *k*_3_ a trimolecular process, for example, Auger recombination involving electron and hole and a third free carrier where two of the particles lose energy with respect to each other while a third particle gains energy, and so the total energy is conserved in this three-particle process.

**Figure 3:**
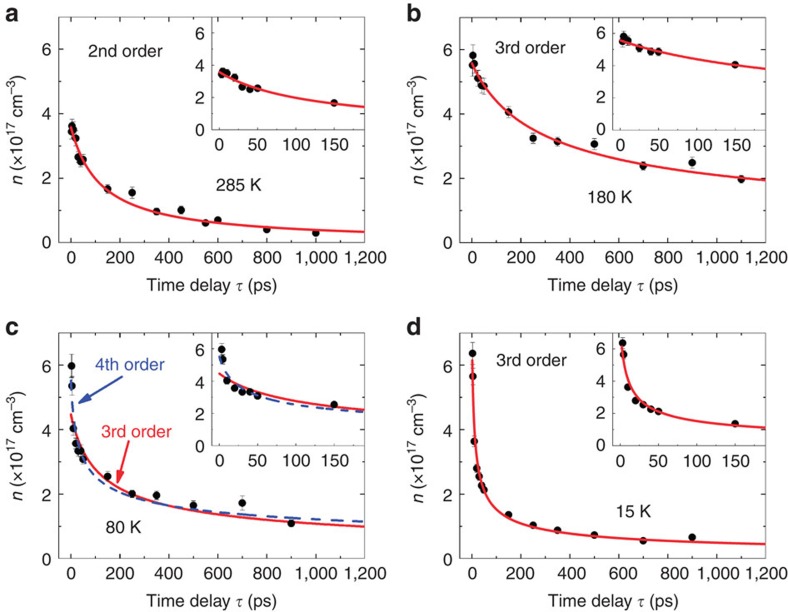
Decay kinetics of the free-carrier density *n*. Free-carrier density *n* obtained from Drude–Smith fits, plotted against pump-probe time delay *τ*, for (**a**) 285 K, (**b**) 180 K, (**c**) 80 K and (**d**) 15 K. Lines are fits of the data to the respective kinetic orders, using the weighted least squares method. The insets show the decrease of carrier density at earlier time delays. The recombination kinetic order evolves from second order at room temperature to third order at 15 K. The error bars of the data points are the standard error of the mean obtained by fitting the complex conductivity spectra to the Drude–Smith model.

Figure 3 shows that the experimental *n* versus *τ* relation represents a temperature-dependent change of recombination kinetics. The data for 285 K are best fitted to an effective second-order behaviour, while the data at 15 K follow third order. At 180 K, the data are equally well fitted by third-order kinetics. Interestingly, at 80 K, the kinetics appear to be fourth order. These results are reproducible from two other samples and reflect incomplete transition from the tetragonal high temperature to the orthorhombic low-temperature crystal structure due to the constraints given by the disordered inhomogeneous film.

It is extremely unlikely to have four entities appearing at the same location and at the same time for a relatively long period of time (1 ns). The fact that the kinetics appears to be higher order at 80 K is a possible indication of more complicated kinetics occurring in the CH_3_NH_3_PbI_3_ thin film. It is often the case in kinetics that one reaction order or summation of orders cannot fully describe the data, and multistep mechanisms were typically involved. For example, in our case suppose that initially (<10 ps) there is, as one step, a very fast conversion of some electrons and holes to excitons or to trap filling, and thereafter (10–1,000 ps) there is an equilibrium between them plus a slower decay by recombination via radiative and non-radiative decay. Such a mechanism cannot be represented by the sum of first-, second- and third-order terms. Instead, one writes a mechanism and obtains a kinetic equation that is more complex. What we have done in our work is merely to discern whether there is an ‘effective' order that dominates the free-carrier recombination process within 1 ns (see Supplementary Discussion for a full statistical analysis of the fits to various orders). Our conclusions are also supported by the corresponding plots of 1/*n*^2^ and 1/*n* versus *τ* given in the Supplementary Discussion. The rate constants from the fits to the experimental data are summarized in Table 1. Note that, in contrast to Wehrenfennig *et al*.^8^, where the product *n*(*τ*) *μ*(*τ*) was fitted to the rate equation assuming *μ*(*τ*) to be *τ* independent, we directly fitted *n*(*τ*) to the rate equation to obtain the rate constants. The increase of carrier mobility and concurrent decrease of carrier density with increasing time delay, was also observed via TRTS in bulk GaAs^19^.

### Quantum yield calculation

The quantum yield of the free-carrier generation is given by *φ*=*n*_0_/*n*_photon_, where the initial photogenerated carrier density *n*_0_ at each temperature results from the fit of the carrier density *n*(*τ*) to the rate equation (Equation 2). The density of absorbed photons is 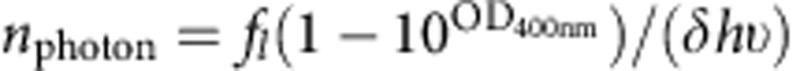, where *f*_*l*_ is the incident laser fluence (∼27 μJ cm^−2^), 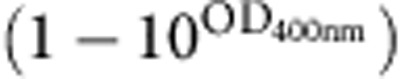 is the fraction of the incident pump power absorbed by the sample and *hν*=3.1 eV the photon energy. The penetration depth *δ* of the 400-nm pump beam is *δ*=1/*α*_400 nm_=69 nm. For an average sample thickness *d*=230 nm, the absorption coefficient *α*_400 nm_=1.45 × 10^5^ cm^−1^ follows from the optical density OD_400 nm_=1.45 taken from Supplementary Fig. 1. As a caveat, we mention that the spectra in Supplementary Fig. 1 are not corrected for scattering and reflection. Therefore, in principle, the OD_400 nm_ represents an upper limit of the true absorbance. However, the effects of scattering and reflection on the absorption spectra are minor: even if OD_400 nm_ in Supplementary Fig. 1 would be overestimated by a factor of 2, the quantum yield would increase by only 20% (see Supplementary Discussion). We add in passing that our value of *α*_400 nm_ is similar to that given in Xing *et al*.^20^ for a thin perovskite film on quartz.

Now that we have apart from the carrier mobility (1+*c*_1_)*μ* and the second-order rate constant *k*_2_, an estimate of the quantum yield *φ* for carrier formation, we can compare our results at 285 K with the recent TRTS papers^8^,^9^ where the products *φμ* and *φk*_2_ were determined in room-temperature measurements. From Fig. 2 and Table 1, we obtain *φμ*(1+*c*_1_)=11 V^−1^ s^−1^ cm^−1^ and *φk*_2_=11 × 10^−10^ cm^3^ s^−1^. These data compare well with the corresponding values of 8.2 V^−1^ s^−1^ cm^−1^ and 9.2 × 10^−10^ cm^3^ s^−1^ in Wehrenfennig *et al*.^8^ where the support was mesoporous alumina. In another TRTS work^9^, similar values for *φμ* and *φk*_2_ are reported for perovskite films on alumina, but also for neat perovskite films. The qualitative independence of *φμ* and *φk*_2_ of the nature of support is most relevant for the production of perovskite-based solar cells. It is rewarding to see that the independently determined quantum yield, mobility and recombination rate of charge carriers in this paper are in agreement with other TRTS works where only the products *φμ* and *φk*_2_ could be given.

### Temperature-dependent kinetic orders

A key result in Table 1 is that at room temperature the decay of charge carriers follows second-order kinetics and that it switches to third order at lower temperatures. Second-order free-carrier recombination kinetics was also observed in room-temperature time-resolved photoluminescence and transient absorption data^21^. As the radiative lifetime for an absorber with a large transition moment is expected to be in the range of nanoseconds (>5 ns for CH_3_NH_3_PbI_3_ thin films)^20^,^22^, half-lives of the carriers of the order of 100 ps point to a recombination process that is predominantly non-radiative. This second-order process is an electron transfer reaction of the charge neutralization type. Its treatment involves taking into account the interaction of the electrons and holes with each other and with the environment before the electron transfer, and the state of the entire system following it. In the polar environment of the perovskite lattice determined by the anionic and cationic species (I^−^, Pb^2+^ and CH_3_NH_3_^+^), the reorganization energy for the electron transfer might be fairly large^23^, large enough so that the system is not too deep in the ‘inverted region' and thus permits the second-order electron transfer to occur. (We add in passing that an electron transfer rate is reduced when in the inverted region^24^ the standard free energy of reaction, that is, the driving force is much larger than the reorganization energy). Recently, also in visible-pump/visible-probe experiments, second-order kinetics was reported for the recovery of the ground-state absorption of a CH_3_NH_3_PbI_3_ perovskite film on excitation^25^. A challenge in those optical pump-probe experiments is, however, to identify the nature of the interacting particles that are not necessarily free charges.

As the temperature is lowered, the decay kinetics of the free charge carriers changes from second to third order. As the half-lives are still in the 100-ps range, non-radiative decay channels have to be considered. As such, third-order kinetics in semiconductor physics is not new. For instance, three-body recombination was seen in nanoparticles^26^,^27^. However, the temperature-dependent change of mechanism may be specific for perovskite-type solids. In the present case, the temperature-dependent change of mechanism may reflect a thermally activated second-order process, while the temperature coefficient of the third-order process is clearly negative. The rate constant *k*_3_ increases with decreasing temperature from 80 to 15 K (Table 1), even though the mobility, as shown in Fig. 2, is relatively insensitive to both temperature and phase transition at larger time delays. Since the reaction at the three lower temperatures follows third-order kinetics, it is not a radiative process. It is a third-order Auger process in which two particles recombine, while the third one carries away the excess energy. One possibility to explain the pronounced temperature dependence of *k*_3_ in the orthorhombic phase is that the two free charge carriers recombine to form an exciton that requires relatively little energy loss of the order of 40 meV^11^. In such a case, it would be possible to explain the negative temperature coefficient of *k*_3_, since the smaller the thermal energy *k*_B_*T*, the smaller the energy needed to be disposed and the faster the recombination reaction^28^. A somewhat analogous process with a negative temperature coefficient of a ‘three-body' process is seen in the combination reaction of atomic and molecular species in the gas phase and has been explained in terms of easier energy disposal when the excess energy (approximately *k*_B_*T*) is small^29^.

### Diffustion length determination

**Figure 4:**
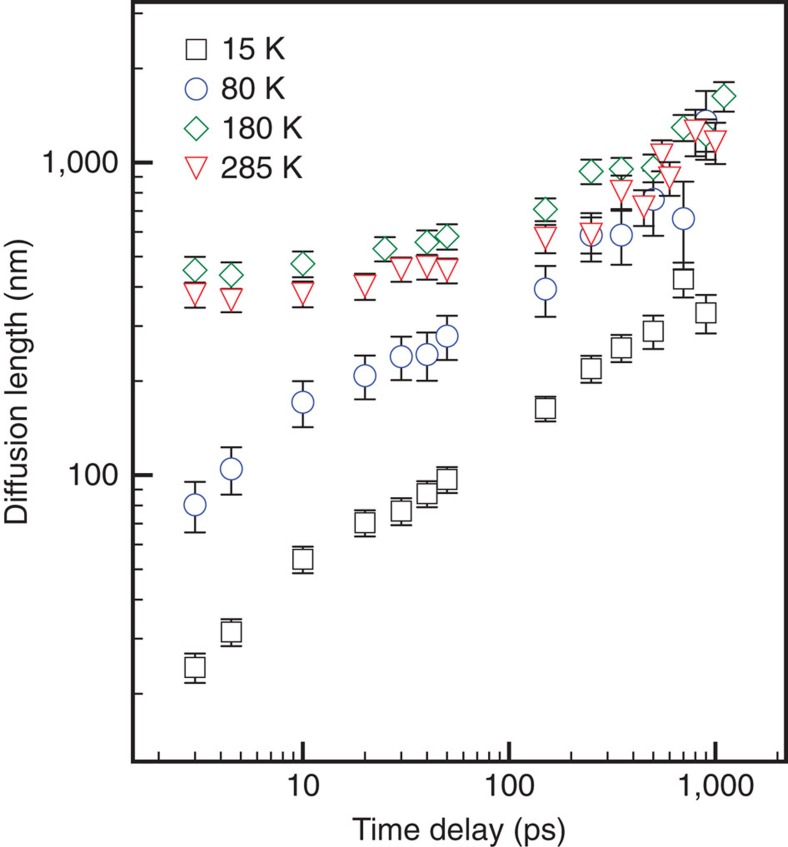
Long diffusion length of perovskite at different pump-probe delay times. At both 180 and 285 K, the diffusion length is of the order of 1 μm, which is larger than the sample thickness of 230 nm. As the temperature is reduced below the tetragonal-to-orthorhombic phase transition, the diffusion length decreases significantly as Auger recombination (via *k*_3_) becomes stronger. The error bars of the data points are the standard error of the mean obtained by fitting the complex conductivity spectra to the Drude–Smith model and the definition of the diffusion length (Equation 3).

The recombination rates, together with carrier mobility, allow for the estimate of the diffusion length *L*_D_, defined as


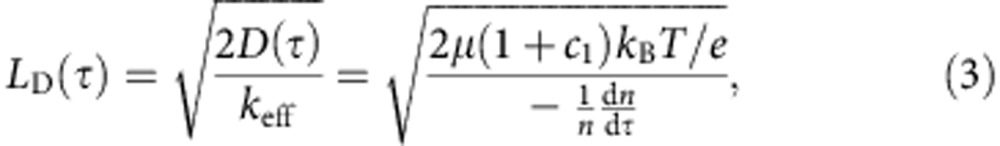


where *D* is the diffusion constant at temperature *T*, *k*_eff_ is the ‘effective first-order' recombination rate constant defined as 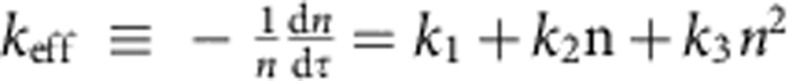. As the numerical factor is determined by the dimensionality of the diffusion, the factor 2 in equation (3) resulting from the Einstein relation accounts for the root mean squared displacement of diffusing charge between parallel planes in a time (*k*_eff_)^−1^. In the present work, *k*_1_=0. For a second-order reaction *k*_eff_=*k*_2_*n*, while for third order *k*_eff_=*k*_3_*n*^2^. The dependence of calculated *L*_D_ values on the pump-probe time delay at various temperatures is plotted in Fig. 4.

At both 180 and 285 K, we obtain *L*_D_ to be of the order of 1 μm, in agreement with other TRTS data^8^,^10^, but longer than those obtained from time-resolved photoluminescence measurements^21^,^30^ that involve electron-donating or electron-accepting contacts. However, at 80 and 15 K, that is, at temperatures below the tetragonal-to-orthorhombic phase transition (as indicated in the absorption spectra of Supplementary Fig. 2), *L*_D_ decreases significantly as Auger recombination (via *k*_3_) becomes stronger. Since one prerequisite for efficient perovskite-based solar cells is a long diffusion length, we can see in Fig. 3 that in our perovskite film the high-temperature tetragonal phase at 285 and 180 K is favoured over the orthorhombic phase at 80 and 15 K.

## Discussion

We have demonstrated the use of picosecond TRTS to independently extract the density and mobility of charge carriers in a perovskite film. Imposed by the disorder of the film, we employed a model that accounts for the effect of backscattering on the free-carrier dynamics. On this basis, we elucidated the nature and the temperature dependence of the free-carrier recombination processes, which directly affect the diffusion length that determines the performance of perovskite-based solar cells.

Note that in our work and analysis, we utilized a pure free-carrier picture, and did not need to invoke the exciton picture. Although excitonic effects are important for optoelectronic applications^31^, they are not relevant to our present work. This is consistent on a few fronts. First, the perovskite exciton-binding energy is commonly agreed to be ∼40 meV (∼10 THz). The number falls well outside our terahertz frequency window, allowing us to omit the contributions from the exciton-binding energy, and the 1*s*-to-2*p* intra-excitonic transition. Second, a recent photoluminescence work found that free-carrier recombination dominates at room temperature, and postulated that the exciton-binding energy should be very small at room temperature^22^, a result corroborated by another high magnetic field report^32^. Third, another recent work reported that the exciton-binding energy is temperature dependent^33^, but once again, these excitonic effects fall outside our terahertz frequency window.

Experimental concepts along the lines described in the present work can be used to explore different organic/inorganic perovskite films, with different morphologies and deposited on different supporting substrates. A recent work showed that carrier radiative recombination rate depends sensitively on film morphology^34^. On elucidating their features in charge carrier density and recombination as well in charge carrier mobility, perovskite films with high performance can be screened. The higher the mobility, the more successfully the charge carriers can travel to the collecting electrodes. In some materials, migration by trapping and detrapping may be important, in which the mobility may have a significant temperature dependence, unlike its weak dependence in the experiments presented in this paper. Our data show that, together with disorder, crystal structure is another crucial factor for the performance of CH_3_NH_3_PbI_3_ solar cells by influencing the Auger recombination process. The values of the free-carrier density, mobility and hence diffusion length given in this paper tend to favour solar cell performance at lower temperatures as long as the sample remains in the tetragonal phase. This suggests the interesting possibility that perovskite-based solar cells could operate well not just on the surface of the earth but also at high altitudes pertaining to airplanes and satellites.

## Methods

### Sample fabrication

Films of nanocrystalline CH_3_NH_3_PbI_3_ (thickness ∼230 nm) on z-cut quartz are grown by a standard solution-based single-step protocol. Z-cut quartz substrates (10 mm × 10 mm × 1 mm) were cleaned thoroughly in HellmanexII solution (1%V/V), deionized water, acetone and isopropanol for 15 min each in an ultrasonic bath. Immediately before deposition of the photovoltaic precursor solution, the substrates were subjected to an air plasma treatment for 5 min and subsequently transferred to a nitrogen glove box. The CH_3_NH_3_I and PbI_2_ components were dissolved in stoichiometric ratio to form the precursor solution (30 wt% in dimethylformamide) for spin coating, followed by heating on a hotplate at 100 °C. The thickness of our final polycrystalline CH_3_NH_3_PbI_3_ film is (230±10) nm. Note that our work was performed on perovskite samples prepared using the single-step method, instead of the more commonly used two-step approach to overcome limitations imposed by poor morphology. We had first verified that the one-step processing technique we used is more straightforward than any of the two-step processes, and that it did not severely limit the solar cell performance (see Supplementary Discussion). Therefore, as our first attempt, we decided to go for the simplest-yet-still-reliable process in preparing our samples. Comparative study on films prepared from various processing techniques is currently being performed.

### Time-resolved terahertz spectroscopy

The perovskite sample is excited by 400-nm (3.1 eV) optical pump pulses that contain about 1.5 eV excess energy above the exciton threshold located at 750 nm. After a well-defined pump-probe time delay *τ*, a terahertz probe pulse arrives at the sample. From the transmitted terahertz electric field *E*(*t*,*τ*) and the photoinduced change in terahertz electric field Δ*E*(*t*,*τ*), we extract the complex photoinduced change in the complex optical conductivity 
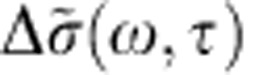
 (referred to as photoconductivity throughout this paper) as a function of *τ* (Fig. 1a,b).

### Online content

More detailed descriptions of temperature-dependent absorption spectra, experimental set-up, TRTS data analysis, rate equation analysis the calculation of quantum yield and diffusion length are available in the online version of the paper; references unique to these sections appear only in the online paper.

## Additional information

**How to cite this article:** La-o-vorakiat, C. *et al*. Elucidating the role of disorder and free-carrier recombination kinetics in CH_3_NH_3_PbI_3_ perovskite films. *Nat. Commun.* 6:7903 doi: 10.1038/ncomms8903 (2015).

## Supplementary Material

Supplementary Information Supplementary Figures 1-8, Supplementary Discussion, Supplementary Methods and Supplementary References

## Figures and Tables

**Table 1:** Temperature-dependent free-carrier recombination kinetics.

***T*** **(K)**	**Order M**	**Rate constants**				
		***k***_**1**_ **(10**^**−12**^** s**^**−1**^**)**	***k***_**2**_ **(10**^**−8**^** cm**^**3**^** s**^**−1**^)	***k***_**3**_ **(10**^**−26**^** cm**^**6**^** s**^**−1**^)	***k***_**4**_ **(10**^**−43**^** cm**^**9**^** s**^**−1**^)	***φ***
15	3	0.04%	0.05%	21±2	—	0.10±0.01
80	4*	0.2%	0.2%	0.3%	1.8±0.3*	0.07±0.01
	3†	0.01%	1.3%	0.34±0.03	—	0.06±0.01
180	3‡	0.1%	0.3%	0.91±0.06‡	—	0.07±0.01
285	2	0.1%	2.2±0.2	—	—	0.05±0.01

The insignificant rate constants are presented as relative contributions (in percentages) to the dominant decay kinetics. The relative contribution of the *m*^th^-order kinetics is measured from the time average of 
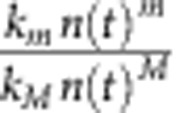
 where *M* is the dominant kinetic order. The recombination switches from second order at room temperature to third order at 15 K. *φ* is the quantum yield of free charge carriers defined as the number of free carriers generated per incident photon.

^*^The kinetics appears to be an ‘unphysical' high order (fourth order), and we ascribe it to a multistep mechanism.

^†^The kinetics is a more physical third order if we ignore the first two data points, that is, fit from 10 ps onwards.

^‡^A single fourth-order kinetics can also fit the data well. We obtain *k*_4_=(2.8±0.2) × 10^−44^ cm^9^ s^−1^ with *φ*=0.08±0.01.
