# Corrigendum: Elucidating the role of disorder and free-carrier recombination kinetics in CH_3_NH_3_PbI_3_ perovskite films

**DOI:** 10.1038/ncomms11054

**Published:** 2016-03-10

**Authors:** Chan La-o-vorakiat, Teddy Salim, Jeannette Kadro, Mai-Thu Khuc, Reinhard Haselsberger, Liang Cheng, Huanxin Xia, Gagik G. Gurzadyan, Haibin Su, Yeng Ming Lam, Rudolph A. Marcus, Maria-Elisabeth Michel-Beyerle, Elbert E. M. Chia

Nature Communications 6: Article number: 8903 10.1038/ncomms8903 (2015); Published: 07302015; Updated: 03102016

This article contains errors in the units used for carrier mobility. In Fig. 2a–d, the units on the *y* axis should be ‘cm^2^V^−1^s^−1^' not ‘V^−1^s^−1^cm^−1^'. Similarly, the second and third sentences of the second paragraph of the ‘Quantum yield calculation' section should read ‘From Fig. 2 and Table 1, we obtain *ϕμ*(1+*c*_1_)=11 cm^2^V^−1^s^−1^ and *ϕk*_2_*=*11 × 10^−10^ cm^3^s^−1^. These data compare well with the corresponding values of 8.2 cm^2^V^−1^s^−1^ and 9.2 × 10^−10^ cm^3^s^−1^ in Wehrenfennig *et al*.^8^ where the support was mesoporous alumina.'

