# Supplementary material for: Elucidating the role of disorder and free-carrier recombination kinetics in CH3NH3PbI3 perovskite films
Source: Nat Commun. 2015 Jul 30;6:7903. doi: 10.1038/ncomms8903 (PMC4532878; doi:10.1038/ncomms8903)
Supplement: Supplementary Information — Supplementary Figures 1-8, Supplementary Discussion, Supplementary Methods and Supplementary References [file ncomms8903-s1.pdf]

Supplementary Figures

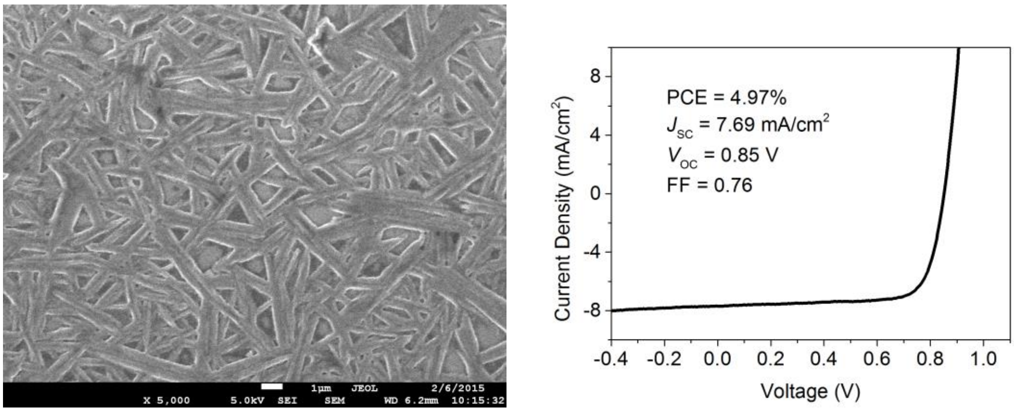

Supplementary Figure 1 | Sample morphology and device performance. Left = SEM micrograph of our $CH_3NH_3PbI_3$ film, showing its morphology. Right = Data showing power conversion efficiency of our thin film.

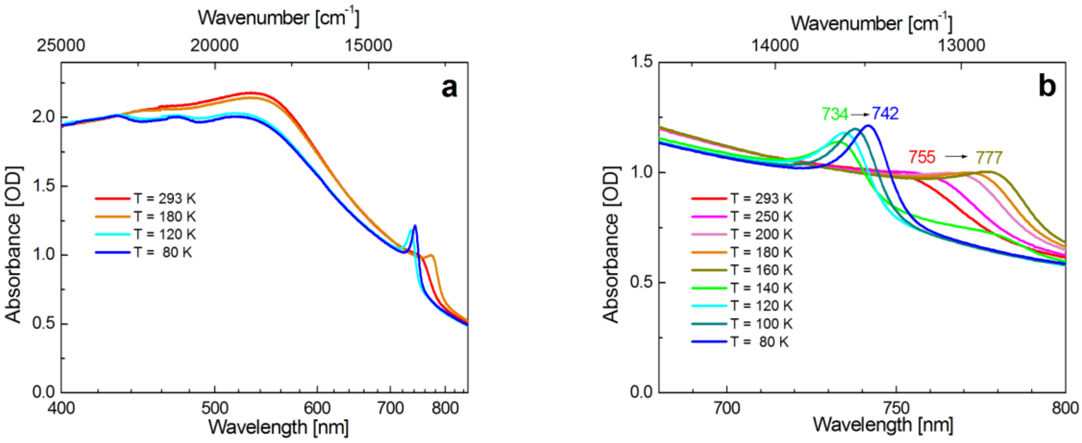

Supplementary Figure 2 | Absorption spectra, of $CH_3NH_3PbI_3$ thin film at various temperatures. (**a**) Spectra from 400 nm – 850 nm. (**b**) Spectra from 680 nm – 850 nm to show, in greater detail, the shifting of absorption peaks with temperature.

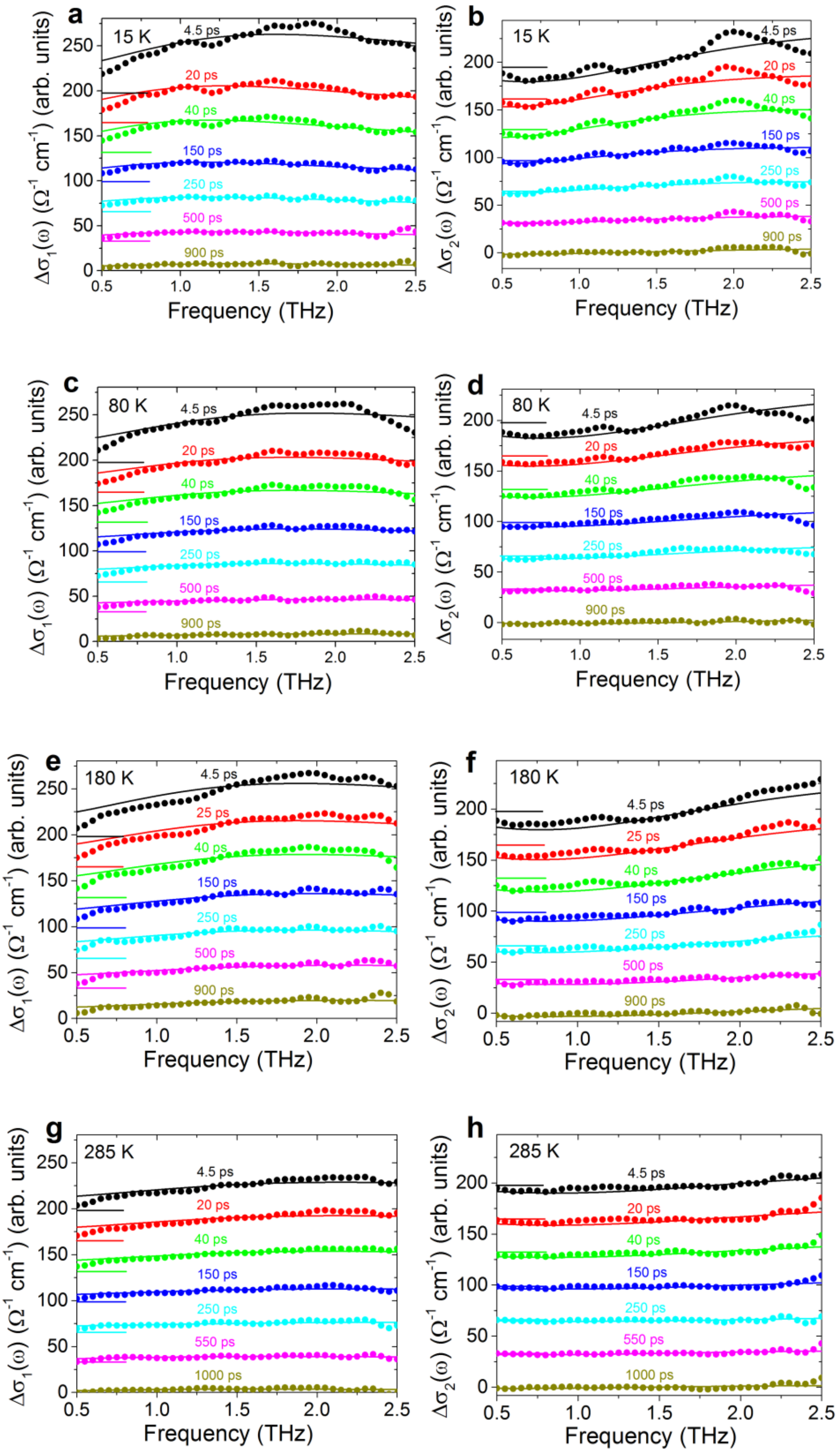

Supplementary Figure 3 | Complex THz photoconductivity ($\Delta\sigma_1 + i\Delta\sigma_2$) at selected time delays at (a, b) 15 K, (c, d) 80 K, (e, f) 180 K and (g, h) 285 K. Data points are in circles and the fits to Drude-Smith model (Equation 1 of the main text) are in dashed lines. Curves are shifted vertically (horizontal lines) for clarity.

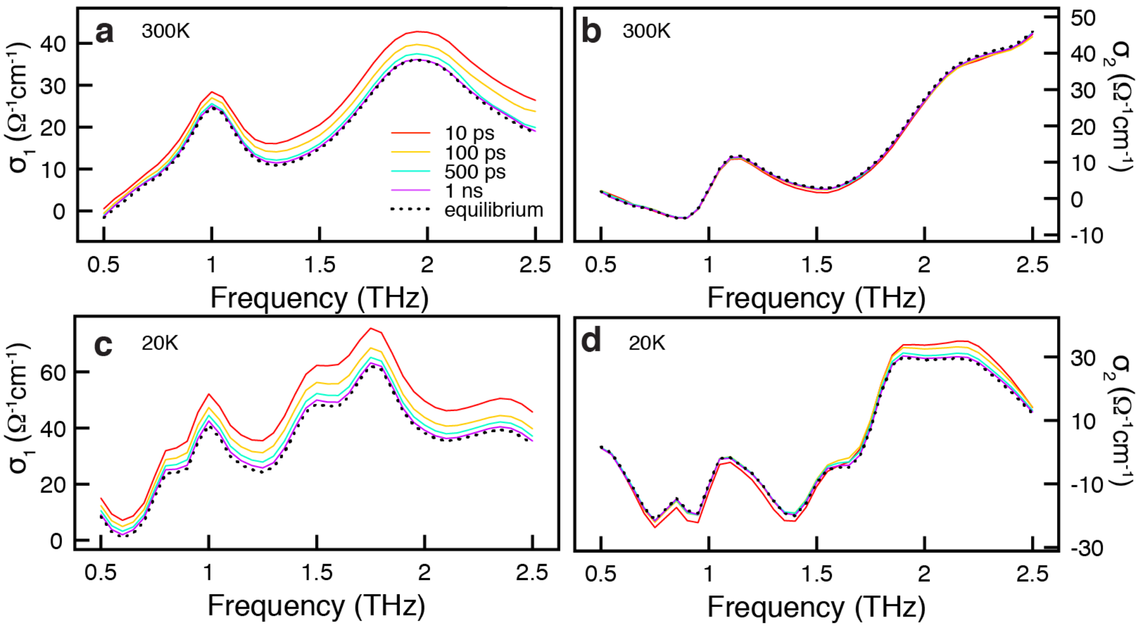

Supplementary Figure 4 | THz conductivity of $CH_3NH_3PbI_3$ at (a, b) 300 K and (c, d) 20 K, at different pump-probe time delays, showing the phonon-mode resonances.

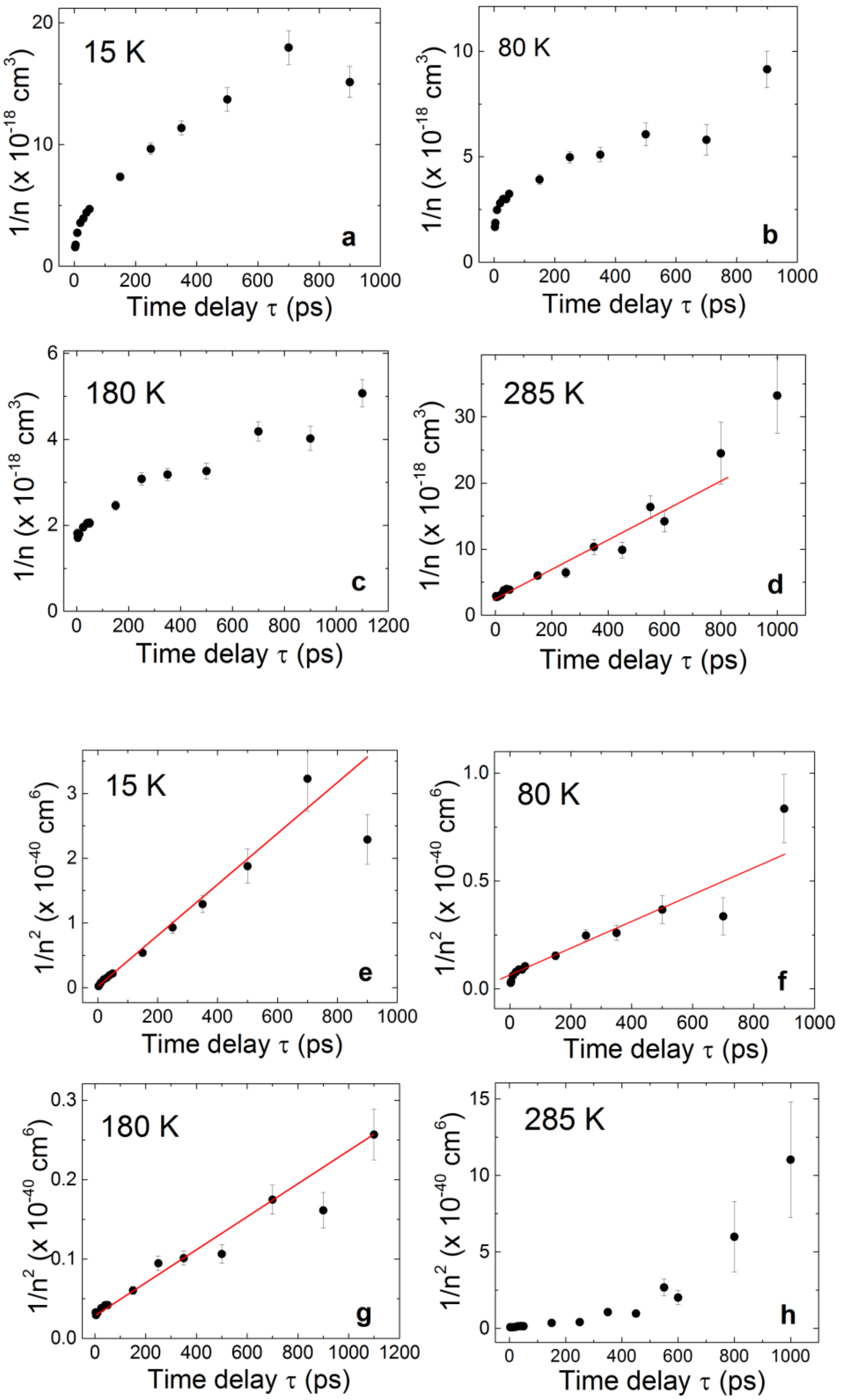

Supplementary Figure 5 | Temporal evolution of carrier density. (**a**–**d**) Inverse carrier density $(1/n)$, and (**e**–**h**) inverse-square carrier density $(1/n^2)$, plotted against pump-probe time delay τ, at 15 K, 80 K, 180 K, and 285 K. The solid lines are guide to the eye to the linear regions, showing that recombination is primarily governed by third-order processes at 15 K, 80 K and 180 K, and second-order processes at 285 K. The error bars of the data points are the standard errors obtained by fitting the complex conductivity spectra to the Drude-Smith model.

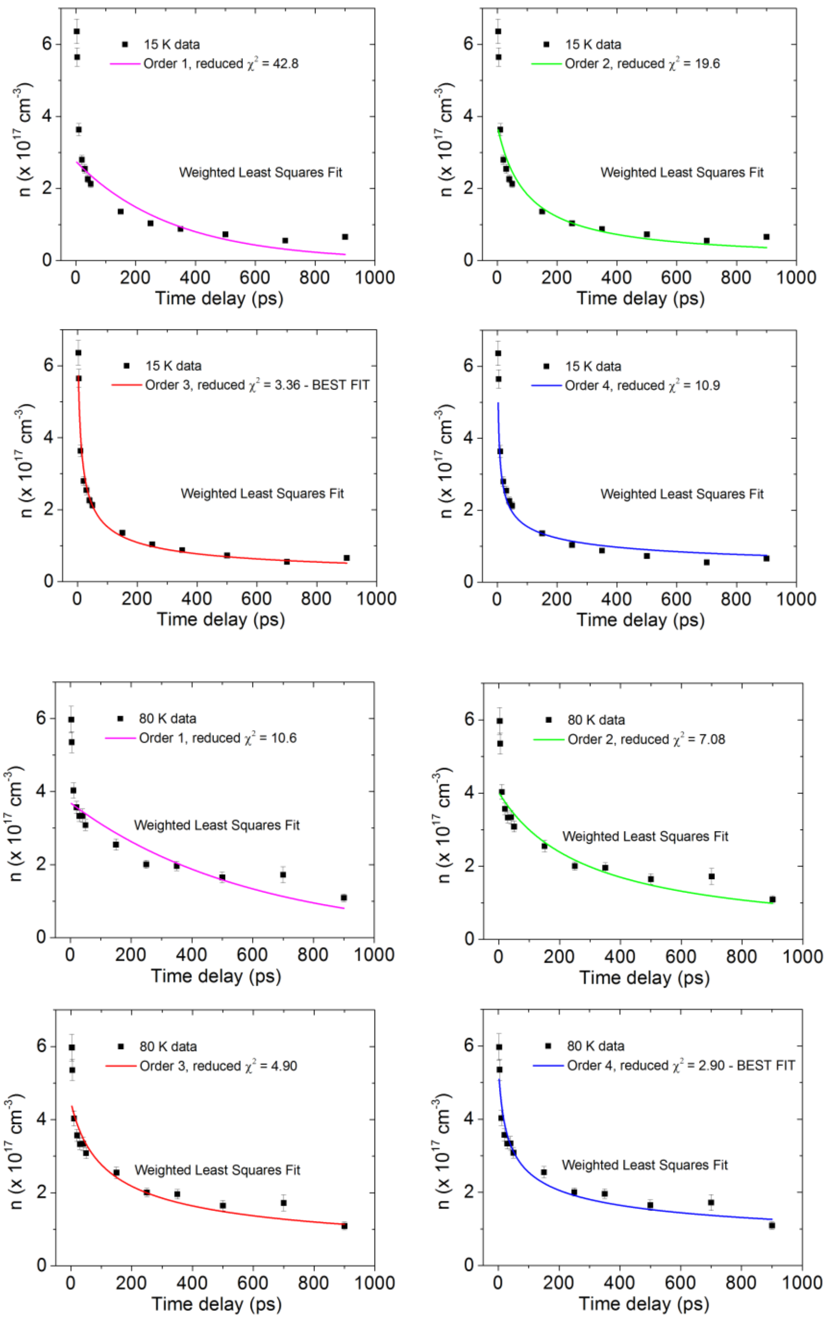

Supplementary Figure 6 | Fitting the temporal evolution of carrier density to the rate equation of various orders. The solid lines are weighted least-squares fits to the rate equation (Equation 1), assuming one dominant order. The results show that recombination kinetics is effectively third order at 15 K, fourth order at 80 K, third or fourth order at 180 K, and second order at 285 K. Results on two other samples show the same behavior at 15 K, 80 K and 285 K, while at 180 K third order kinetics fit the data better than fourth order. The reduced χ^2 values were obtained from fits to the entire time delay range, i.e. down to 3 ps. The error bars of the data points are the standard errors obtained by fitting the complex conductivity spectra to the Drude-Smith model.

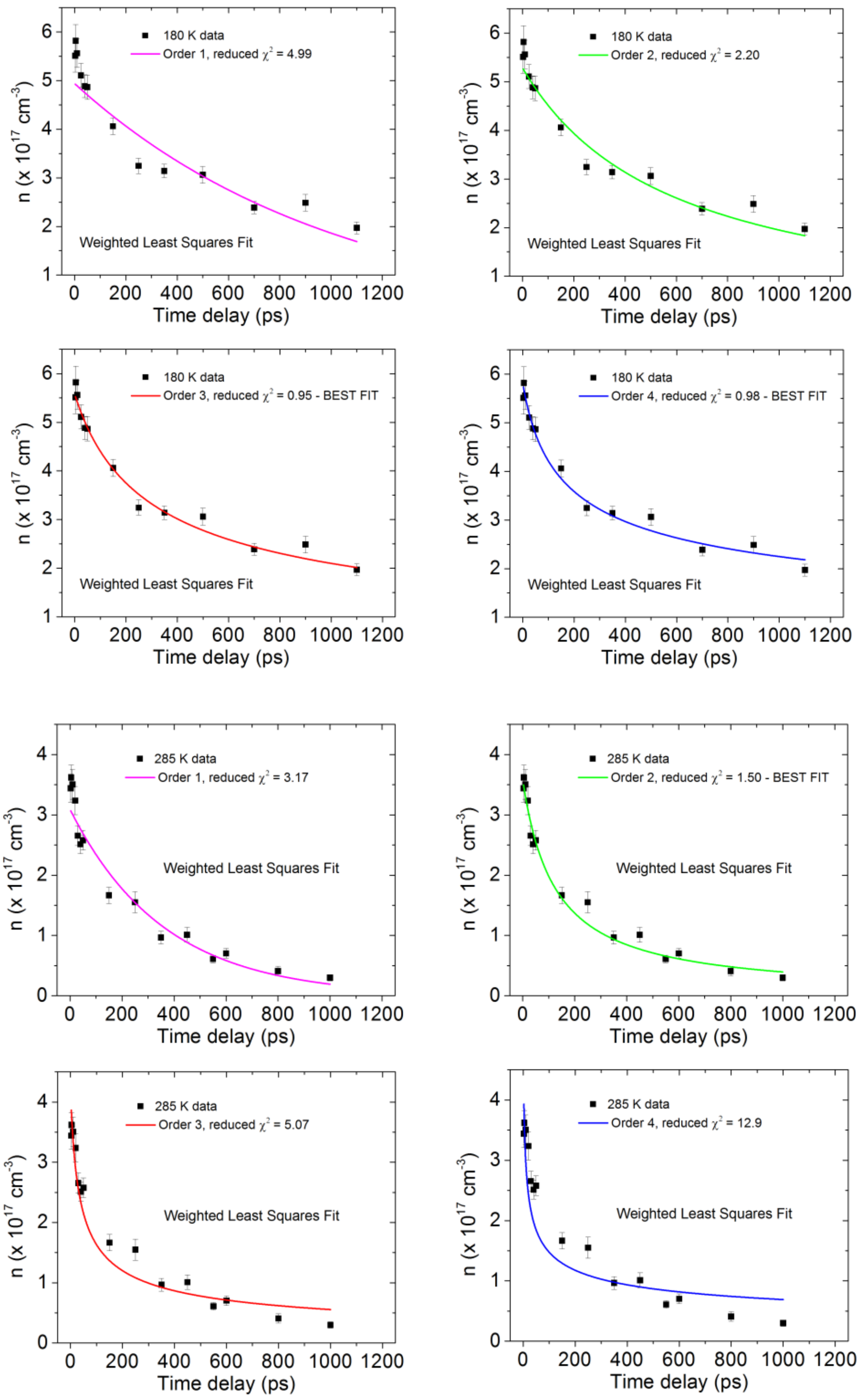

Supplementary Figure 6 (cont.) | Fitting the temporal evolution of carrier density to the rate equation of various orders.

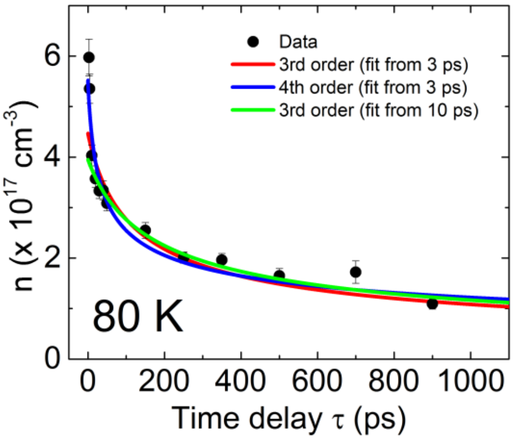

Supplementary Figure 7 | Various rate equation fits at 80 K. Third and fourth order fits starting from different initial times. The error bars of the data points are the standard errors obtained by fitting the complex conductivity spectra to the Drude-Smith model.

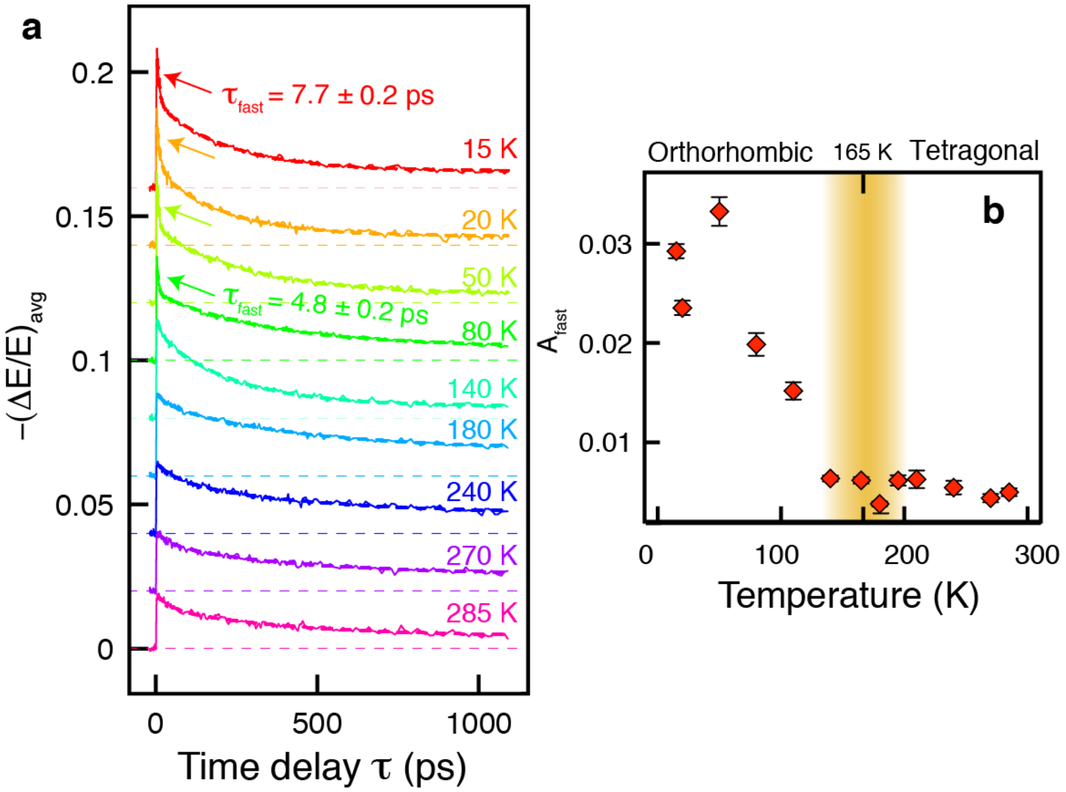

Supplementary Figure 8 | Frequency-integrated time-resolved terahertz spectroscopy (TRTS). (**a**) Frequency-integrated THz transient $\frac{\Delta E_{peak}(\tau)}{E_{peak}}$, of $CH_3NH_3PbI_3$, as a function of pump-delay (τ) and sample temperature. The fast relaxation of the TRTS signal are highlight by the arrows. (**b**) The enhancement of fast decay amplitude suggests the increasing contribution of the Auger mechanism at low temperatures. The yellow window around 165 K represents the width of the structural phase transition in our film samples. The error bars of the data points are the standard errors from the exponential fittings.

Supplementary Discussion

Potential Influence of Scattering and Reflection on the Absorption Spectra, Carrier Density, Carrier Mobility, Diffusion Length and Quantum Yield

As the absorption spectra were measured in transmission, they may include reflection and scattering besides absorption. Therefore, the absorption spectra in Supplementary Figure 2 represent an upper bound of the absorbance.

In order to assess the potential influence of scattering and reflection on the absorption spectra, even large contributions of such effects will have only minor consequences. Let us assume that the true optical density OD_{400nm} of the film at the wavelength of excitation is in reality smaller by a factor of $\boldsymbol{x = 2}$ than the one stated in Supplementary Figure 2. This uncertainty will result in an twofold increase in the optical penetration depth $\boldsymbol{\delta}$, a twofold decrease of the carrier density $\boldsymbol{n(\tau)}$. *However, the fitted Drude-Smith mobility* $\boldsymbol{(1 + c_1)\mu}$ *and the diffusion length* $\boldsymbol{L_D}$ *are not affected.* By fitting $\boldsymbol{n(\tau)}$ to the rate equation (Equation 1), the value for the fitted second-order rate constant $\boldsymbol{k_2}$ prevailing at room temperature will double, while the third order rate constant $\boldsymbol{k_3}$ (predominant at 180 K, 80 K and 15 K) will increase by a factor 4.

The quantum yield of the free carrier generation is given by $\phi = n_0/n_{photon}$, where the initial photogenerated carrier density n_0 at each temperature results from the fit of the carrier density $n(\tau)$ to the rate equation. The density of absorbed photons is $n_{photon} = f_l(1 - 10^{OD_{400nm}})/(\delta h\nu)$, where f_l is the incident laser fluence (~$27\ \mu J\ cm^{-2}$), $(1 - 10^{OD_{400nm}})$ is the fraction of the incident pump power absorbed by the sample, and $h\nu$ = 3.1 eV the photon energy. The penetration depth δ of the 400-nm pump beam is $\delta = 1/\alpha_{400nm} = 69$ nm. For an average sample thickness $d = 230$ nm, the absorption coefficient $\alpha_{400nm} = 1.45 \times 10^5\ cm^{-1}$ follows from the optical density OD_{400nm} = 1.45 taken from Supplementary Figure 2. Since the spectra in Supplementary Figure 2 are not corrected for scattering and reflection, in principle, the OD_{400nm} represents an upper limit of the true absorbance. However, the effects of scattering and reflection on the absorption spectra are minor: even if OD_{400nm} in Suplementary Figure 2 would be overestimated by an unrealistic factor of 2, the fitted initial carrier density n_0 would be reduced by a factor of 2, the penetration depth doubled, and the absorbed photon number 16% smaller [$(1 - 10^{-0.72})$ instead of $(1 - 10^{-1.45})$]. The corrected quantum yield ϕ at 285 K will then be 0.056, instead of 0.047, i.e. the quantum yield of charge generation in Table 2 would be overestimated by only 20%. We mention in passing that experimental corrections for scattering[1,2] and for reflection[3] also involve uncertainties caused by approximations and errors of parameters. Also our value of α_{400nm} is similar to that given in Xing *et al.*[1] for a thin perovskite film on quartz ($1.5 \times 10^5\ cm^{-1}$)

Phonon Mode Resonances in $CH_3NH_3PbI_3$

The THz conductivity of $CH_3NH_3PbI_3$ is dominated by phonons (Supplementary Fig. 4). We detect two strong phonon peaks at 300 K. By use of two Lorentz modes, we are able to obtain resonance frequencies at 0.95 and 2.0 THz, which agree with previous THz studies[4,5]. At low temperatures, the two peaks split into four peaks at 0.75, 0.95, 1.45 and 1.80 THz. The change in spectral feature reflects the structural phase transition from tetragonal to orthorhombic that occurs near 165 K[6].

Rate Equation Analysis

In the main text, we used the rate equation

$$\frac{dn(\tau)}{d\tau} = -k_1 n - k_2 n^2 - k_3 n^3 \quad (1)$$

to describe the recombination dynamics up to third-order process. We also plotted $1/n(\tau)$ and $1/n^2(\tau)$ versus τ, at 15 K, 80 K, 180 K and 285 K (see Supplementary Figure 5), to determine which order process dominates the recombination process. If $1/n(\tau)$ varies linearly with τ, then the recombination is primarily second order. If $1/n^2(\tau)$ varies linearly with τ, the recombination is primarily third order. From Supplementary Figure 5, we can see that at low temperatures, recombination is governed primarily by third-order (e.g. Auger) processes, while at room temperature, it crosses over to second order.

Supplementary Figure 6 show weighted least squares fits of $n(\tau)$ versus τ assuming one dominant order. The results show that recombination kinetics is effectively third order at 15 K, fourth order at 80 K, third or fourth order at 180 K, and second order at 285 K. If we ignore the first two points of the 80 K data, then the recombination kinetics is best fit by third order kinetics. We elaborate on each temperature in the following discussions.

15K and 285K: The analysis in Supplementary Figure 6 confirms that at 15 K, the recombination kinetics is effectively third order. In the same manner, the 285 K kinetics is effectively second order. These conclusions for 285 K and 15 K are corroborated by TRTS data taken on two other samples.

180 K: Third and forth orders kinetics fit the data equally well, hence we present both sets of fitting parameters in Table 1. However, the same measurements performed on two other samples show that third-order kinetics fits the data better than fourth order. We therefore attribute the kinetics at 180 K to the physically more reasonable scenario of third-order recombination (e.g. Auger recombination).

80 K: The analysis at 80 K shows that the recombination order appears to be fourth order. This fourth-order result is reproducible in two other samples we measured. However, if we ignore the first two data points, the recombination becomes third order, which is a more physically possible situation (e.g. Auger recombination).

A fourth order free-carrier recombination was observed in GaN-based light-emitting diodes[7], and was used to explain a drop in LED efficiency for large current-injection density. However, we do not believe that our kinetics at 80 K is fourth order, as it is extremely unlikely to have four entities appearing at the same location and at the same time from 0 to 1 ns. The fact that the kinetics appears to be high order is a possible indication of more complicated kinetics occurring in the $CH_3NH_3PbI_3$ thin film at 80 K. It is often the case in kinetics, that one reaction order or summation of orders cannot fully describe the data, and multistep mechanisms were typically involved. Generally, free carriers can decay directly either by radiative or non-radiative recombination without first forming excitons. Or, they can first form excitons, which can either dissociate to form free carriers again, or recombine (radiatively or non-radiatively). All these different pathways occur with different probabilities, resulting in a complicated set of coupled differential equations.

A possible multistep scenario is, suppose there is initially, as one step, a conversion of some electrons and holes to excitons, and thereafter there is an equilibrium between them plus a slow decay by recombination via radiative and non-radiative decay. Such a mechanism cannot be represented by the sum of a first, second and third order terms. Instead one writes a mechanism and obtains a kinetic equation that is more complex. This may explain our 80 K data, where the initial fast recombination (< 10 ps) cannot be represented by a third-order recombination, while data beyond 10 ps can be represented by third-order recombination (see green curve in Supplementary Figure 7). What we have done in our work is merely to discern whether there is an "effective" order that dominates the free carrier recombination process within one nanosecond.

<u>Recombination Dynamics via Frequency-Integrated Optical-Pump THz-Probe</u>

Since the maximum of L_D is located near 180 K (see Supplementary Figure 5 of main text), we take a closer look at the temperature-dependent dynamics especially across the tetragonal-to-orthorombic structural phase transition temperature near 165 K[8,9,10]. We therefore obtain *frequency-integrated* THz transient data, by measuring the peak amplitude of the THz electric field ($\frac{\Delta E_{peak}(\tau)}{E_{peak}}$) as a function of pump-probe time delay (Supplementary Figure 8a). This method shortens the data acquisition time significantly, allowing us to obtain the data with more time steps and temperature points. However, the ability to disentangle and extract the free-carrier density n is sacrificed because the integrated signal is only proportional the *frequency-integrated* photoconductivity amplitude. We then fit the peak THz electric field with the double-exponential expression

$$\frac{\Delta E_{peak}(\tau)}{E_{peak}} = A_{fast}e^{-\tau/\tau_{fast}} + A_{slow}e^{-\tau/\tau_{slow}} + A_{res}. \quad (2)$$

The fits show that the amplitude of fast-decay component (A_{fast}) is significantly enhanced in the low-temperature orthorhombic phase (Supplementary Figure 8b). This suggests that the fast decay is associated with the Auger recombination mechanism as also found in the TRTS study of ZnO[11]. This Auger mechanism is stronger in the orthorhombic phase than in the tetragonal phase, providing a strong recombination channel during early times (< 10 ps, arrows in Supplementary Figure 8a). In addition, the small values of the third-order recombination rate contant k_3 at 180 K (1.0×10^{-26} $\mathrm{cm^6\ s^{-1}}$) and 285 K (~$0\ \mathrm{cm^6\ s^{-1}}$) are also in agreement with the disappeance of this fast relaxation channel.

Supplementary Methods

Materials

Z-cut quartz substrates (10 x 10 x 1 mm) were cleaned thoroughly in Hellmanex®II solution (1%V/V), DI water, acetone and isopropanol for 15 minutes each in an ultrasonic bath. Immediately before deposition of the photovoltaic precursor solution, the substrates were subjected to an air plasma treatment for 5 minutes and subsequently transferred to a nitrogen glove box. The CH_3NH_3I and PbI_2 components were dissolved in stoichiometric ratio to form the precursor solution (30 wt% in dimethylformamide) for spin coating, followed by heating on a hotplate at 100 °C. The thickness of our final polycrystalline $CH_3NH_3PbI_3$ film is (230 ± 10) nm.

Sample Morphology and Device Performance

The left image on Supplementary Figure 1 shows the top-view SEM micrograph. It demonstrates the typical morphology of our $CH_3NH_3PbI_3$ film. Similar morphologies have been obtained by other groups as well, i.e., Jeon *et al.*[12] and Huang *et al.*[13]. The fast solvent evaporation resulted in a rapid perovskite crystal formation on flat substrate in the form of micron-sized dendritic crystallites. The single-step fabrication method, which was employed in our study, tends to generate film with sub-optimal morphology. However, we have first established that devices prepared with these perovskite films could still result in acceptable power conversion efficiency of around 5% (right image). In view of the simplicity involved in sample preparation, we decided to opt for the single-step processing. It is our intention to study the films prepared with other techniques in the near future.

Absorption Spectrum

The steady-state absorption spectra presented in Supplementary Figure 2 were measured on perovskite films in transmission (Varian Cary 100 Bio UV-VIS spectrophotometer). The absorption spectrum shows a monotonic increase from the lowest exciton transition at 750 nm (~1.5 eV) up to 500 nm and flattens off thereafter. It shows no temperature dependence at 400 nm, which is relevant for the interpretation of low-temperature measurements. Upon cooling below 160 K a new absorption peak at about 735 nm gradually grows at the expense of the long wavelength band and, similar to the 755 nm band before, also red-shifts as the temperature is further decreased. We associate this spectral change with the tetragonal-to-orthorhombic structural phase transition which is observed in single crystals[14] at 162.5 K. In contrast, the solution-processed perovskite film in this study displays a phase transition over an approximate 20 K temperature window between 160 K and 140 K. This sluggish phase transition is attributed to constraints imposed by the grain boundaries in the nanocrystalline film and its disorder.

Experimental Setup: Time-Resolved THz Spectroscopy (TRTS)

Femtosecond pulses are provided by a standard regenerative Ti:sapphire-laser amplifier system (1 kHz, 50 fs, 780 nm, 2.5 mJ). Part of the pulse energy is used for the generation and detection of THz pulse. We generate THz pulses by optical rectification method from a ZnTe crystal (1-mm thick) by use of a laser pulse energy of 150 μJ, providing a usable THz bandwidth up to 2.5 THz. The THz beam is focused onto the sample with a parabolic mirror down to the spot size of 2.0 mm (measured by the knife-edge method). After passing through the sample, we collimate and focus the transmitted THz beam to a second ZnTe crystal (0.5-mm thick) for the detection of the complex THz electric field via electro-optical sampling method. By scanning the delay t between the THz and gate pulses, we obtain the

time-domain profile of the THz pulses. We mount the sample in a cryostat equipped with a vertical motorized stage to move between the $CH_3NH_3PbI_3$ film grown on a bare *z*-cut quartz substrate (sample) and a *z*-cut quartz substrate (reference), to extract the THz conductivity of the sample. The rest of the laser pulse energy is used for optical excitation of the sample. We use a BBO crystal to excite the sample with 400-nm light and a small excitation power (1.7 mW) on a large spot (4-mm diameter) to avoid sample damage (fluence of $27\,\mu\mathrm{J\,cm^{-2}}$). The time delay τ between the 400-nm pump and THz probe pulses is provided by another optical delay stage.

We modulate both the THz and pump arm by two choppers rotating at two different frequencies[15], and demodulate using two lock-in amplifiers, in order to simultaneously obtaine both (a) *transmitted THz electric field* through sample [$E_{sam}(t,\tau)$] and reference [$E_{ref}(t,\tau)$] (THz-arm modulation) and (b) *pump-induced change* in the THz electric field [$\Delta E_{sam}(t,\tau)$] (pump-arm modulation). We obtain the pump-induced transient via the direct measurement of $\Delta E_{sam}(t,\tau)$ due to its superior signal-to-noise ratio.

We also synchronize the time delay between optical pump arm (τ) and the gate arm (t) such that the entire measured THz transient experiences the same delay from the pump pulse[16,17], which is critically important when the pump delay is less than THz pulse duration (< 5 ps).

Data Analysis: Photoconductivity Calculation

In the following, we describe our method to calculate the photoinduced change in complex THz conductivity [$\Delta\tilde{\sigma}(\omega,\tau) = \Delta\sigma_1 + i\Delta\sigma_2$] at time delay τ and angular frequency $\omega = 2\pi f$, from the measurements of the time-domain transmitted electric field $E_{sam(ref)}(t,\tau)$ and photoinduced change in electric field $\Delta E_{sam(ref)}(t,\tau)$, through the sample (reference).

(a) We calculate the $CH_3NH_3PbI_3$ thin-film transmission coefficient *at equilibrium* from the transmitted electric field through the sample ($CH_3NH_3PbI_3$ film on *z*-cut quartz substrate) and reference (bare *z*-cut quartz substrate).

(b) We then extract both equilibrium and transient complex refractive indexes ($\tilde{n} = n + ik$) from the THz transmission functions by numerically solving the equation[18]

$$\tilde{T}(\omega,\tau) = \frac{2\tilde{n}(\tilde{n}_{sub}+1)\exp[\frac{i\omega d(\tilde{n}-1)}{c}]\exp[\frac{-i\omega\Delta L(\tilde{n}_{sub}-1)}{c}]}{(1+\tilde{n})(\tilde{n}+\tilde{n}_{sub})+(\tilde{n}-1)(\tilde{n}_{sub}-\tilde{n})\exp[\frac{2i\omega d\tilde{n}}{c}]}, \quad (3)$$

where $\tilde{n}_{sub}$ is a refractive index of *z*-cut quartz substrate obtained from a separate measurement, d is the film thickness, c is the speed of light in vacuum, and ΔL is the difference in thickness of sample and reference substrates. This equation is the exact formula applied to any thin film on a substrate, which is a generalized version of the more popular equation, $\tilde{T}(\omega,\tau) = \frac{1+\tilde{n}_{sub}}{1+\tilde{n}_{sub}+Z_0\tilde{\sigma}(\omega,\tau)d}$, which only applies to a *thin metallic* film ($\tilde{n} \gg \tilde{n}_{sub} > 1$ and $\tilde{n}\omega d/c \ll 1$) on a substrate. The $\tilde{n} \gg \tilde{n}_{sub}$ assumption fails to hold for our $CH_3NH_3PbI_3$ sample, as the extracted refractive index in the THz range is similar to the refractive index of *z*-cut quartz ($\tilde{n}_{sub} \approx 2.1$ in THz range).

The complex THz conductivity $\tilde{\sigma}_{eff}(\omega,\tau) = \sigma_1 + i\sigma_2$ can then be calculated via $\tilde{n} = \sqrt{\tilde{\varepsilon}}$ and $\tilde{\varepsilon} = 1 + \frac{i}{\omega\varepsilon_0}\tilde{\sigma}$.

(c) We calculate photoinduced changes in THz conductivity: $\Delta\tilde{\sigma}_{eff}(\omega,\tau) = \tilde{\sigma}_{eff}(\omega,\tau) - \tilde{\sigma}_0$. The subscript *eff* refers to an *effective* photoconductivity of the sample, since it is calculated by assuming that the entire film of thickness d is photoexcited. However, in reality, we excite the sample only within its penetration depth δ.

(d) We then correct for this partial excitation across the sample thickness by treating the sample as two dielectric slabs — one is photoexcited with conductivity $\tilde{\sigma}_{exc}$, and the other is unexcited with conductivity $\tilde{\sigma}_0$. Since the conductance (defined as conductivity multiplied by thickness) is additive, we can relate these conductivities as

$$d\,\tilde{\sigma}_{eff} = \delta\tilde{\sigma}_{exc} + (d-\delta)\tilde{\sigma}_0\,.$$

Next we define the photoinduced change in THz conductivity *of the photoexcited layer* $\Delta\tilde{\sigma}$ as $\Delta\tilde{\sigma} \equiv \tilde{\sigma}_{exc} - \tilde{\sigma}_0$. After some algebra, we derive the simple expression

$$\Delta\tilde{\sigma} = \frac{d}{\delta}\Delta\tilde{\sigma}_{eff}, \quad (4)$$

which shows that the *effective* photoconductivity must be scaled up by a factor of $d/\delta = 230/69 = 3.33$ to obtain the actual photoconductivty $\Delta\tilde{\sigma}$. This $\Delta\tilde{\sigma}$ is finally the *frequency-resolved* pump-induced change in THz conductivity mentioned throughout our work.

Supplementary Figure 3 shows the complex THz photoconductivity ($\Delta\sigma_1 + i\Delta\sigma_2$) obtained at selected time delays at 15 K, 80 K and 180 K, plus the corresponding Drude-Smith fits.

We also performed a one-dimensional THz scan by fixing the gate-THz time delay t at the THz peak, then scan only the optical-pump-THz-probe delay τ. This method measures only the *frequency-averaged* pump-induced change in THz transmission, without having any frequency resolution. The advantage of the one-dimensional scan is to significantly reduce the data acquisition time.

Supplementary References

1. G. Xing, N. Mathews, S. Sun, S. S. Lim, Y. M. Lam, M. Gratzel, S. Mhaisalkar, T. C. Sum. Long-range balanced electron- and hole-transport lengths in organic-inorganic $CH_3NH_3PbI_3$. *Science* **342,** 344-347 (2013).
2. S. D. Stranks, G. E. Eperon, G. Grancini, C. Menelaou, M. J. P. Alcocer, T. Leijtens, L. M. Herz, A. Petrozza, H. J. Snaith. Electron-hole diffusion lengths exceeding 1 micrometer in an organometal trihalide perovskite absorber. *Science* **342,** 341-344 (2013).
3. M. Cesaria, A. P. Caricato, M. Martino. Realistic absorption coefficient of ultrathin films. *J. Opt.* **14,** 105701 (2012).
4. C. Wehrenfennig, G. E. Eperon, M. B. Johnston, H. J. Snaith, L. M. Herz. High charge carrier mobilities and lifetimes in organolead trihalide perovskites. *Adv. Mater.* **26,** 1584-1589 (2014).
5. C. Wehrenfennig, M. Liu, H. J. Snaith, M. B. Johnston, L. M. Herz. Charge-carrier

dynamics in vapour-deposited films of the organolead halide perovskite $CH_3NH_3PbI_{3-x}Cl_x$. *Energy Environ. Sci.* **7,** 2269-2275 (2014).
6. Noriko Onoda-Yamamuro, Takasuke Matsuo, Hiroshi Suga. Calorimetric and IR spectroscopic studies of phase transitions in methylammonium trihalogenoplumbates (II). *J. Phys. Chem. Solids* **51,** 1383-1395 (2002).
7. Q. Dai, Q.F. Shan, J. Cho, E. F. Schubert, M. H. Crawford, D. D. Koleske, M.-H. Kim, Y. Park. On the symmetry of efficiency-versus-carrier- concentration curves in GaInN/GaN light-emitting diodes and relation to droop-causing mechanisms. *Appl. Phys. Lett.* **98,** 033506 (2011).
8. M. C. Beard, G. M. Turner, C. A. Schmuttenmaer. Transient photoconductivity in GaAs as measured by time-resolved terahertz spectroscopy. *Phys. Rev. B* **62,** 15764 (2000).
9. A. Poglitsch, D. Weber. Dynamic disorder in methylammoniumtrihalogenoplumbates (II) observed by millimeter-wave spectroscopy. *J. Chem. Phys.* **87,** 6373-6378 (1987).
10. C. C. Stoumpos, C. D. Malliakas, M. G. Kanatzidis. Semiconducting tin and lead iodide perovskites with organic cations: Phase transitions, high mobilities, and near-infrared photoluminescent properties. *Inorg. Chem.* **52,** 9019-9038 (2013).
11. E. Hendry, M. Koeberg, M. Bonn. Exciton and electron-hole plasma formation dynamics in ZnO. *Phys. Rev. B* **76,** 045214 (2007).
12. Ye-Jin Jeon, Sehyun Lee, Rira Kang, Jueng-Eun Kim, Jun-Seok Yeo, Seung-Hoon Lee, Seok-Soon Kim, Jin-Mun Yun, Dong-Yu Kim. Planar heterojunction perovskite solar cells with superior reproducibility. *Sci. Rep.* **4,** 6953 (2014).
13. F. Huang, Y. Dkhissi, W. Huang, M. Xiao, I. Benesperi, S. Rubanov, Y. Zhu, X. Lin, L. Jiang, Y. Zhou, A. Gray-Weale, J. Etheridge, C. R. McNeill, R. A. Caruso, U. Bach, L. Spiccia, Y.-B. Cheng. Gas-assisted preparation of lead iodide perovskite films consisting of a monolayer of single crystalline grains for high efficiency planar solar cells. *Nano Energy* **10,** 10 (2014).
14. T. Ishihara. Optical properties of PbI-based perovskite structures. *J. Lumin* **60&61,** 269-274 (1994).
15. A. J. Frenzel, C. H. Lui, Y. C. Shin, J. Kong, N. Gedik. Semiconducting-to-metallic photoconductivity crossover and temperature-dependent drude weight in graphene. *Phys. Rev. Lett.* **113,** 056602 (2014).
16. M. C. Beard, G. M. Turner, C. A. Schmuttenmaer. Transient photoconductivity in GaAs as measured by time-resolved terahertz spectroscopy. *Phys. Rev. B* **62,** 15764 (2000).
17. J. T. Kindt, C. A. Schmuttenmaer. Theory for determination of the low-frequency time-dependent response function in liquids using time-resolved terahertz pulse spectroscopy. *J. Chem. Phys.* **110,** 8589-8596 (1999).
18. Xingquan Zou, Jingzhi Shang, Jianing Leaw, Zhiqiang Luo, Liyan Luo, Chan La-o vorakiat, Liang Cheng, S A Cheong, Haibin Su, Jian-Xin Zhu, Yanpeng Liu, Kian Ping Loh, A H Castro Neto, Ting Yu, Elbert E M Chia. Terahertz conductivity of twisted bilayer graphene. *Phys. Rev. Lett*, **110,** 067401 (2013).
